# Morphometric and Biochemical Parameters of *Apis mellifera* Workers Fed on Protein Supplement

**DOI:** 10.3390/vetsci13070629

**Published:** 2026-06-27

**Authors:** Rasha S. Sakla, Aida A. Abd El-Wahed, Wael Mahmoud Aboulthana, Sobhia S. Sayed

**Affiliations:** 1 Department of Bee Research, Plant Protection Research Institute, Agricultural Research Centre, Giza 12627, Egypt; rasha.s.sakla@gmail.com; 2 Biochemistry Department, National Research Centre, 33 El Bohouth St. (Former ElTahrir St.), Dokki, Giza 12622, Egypt; wmkamel83@hotmail.com

**Keywords:** honey bees workers, protein diet, glands measurement, biochemical activities, enzyme activity

## Abstract

Nutrition is one of the main challenges facing honey bees, especially during autumn and winter. This work aimed to evaluate the effects of feeding *Apis mellifera* with a protein-enriched diet (YCPC) compared to a control diet. Development of the hypopharyngeal glands (HPGs), dimensions of the second wax mirror, and the stinging apparatus were measured in the two groups. Additionally, key biochemical parameters were assessed in homogenized worker honey bees. No significant differences were observed in hypopharyngeal gland (HPG), acini diameters or surface area, or in the measurement of the stinging apparatus and poison sac, except for a slight decrease in the newly emerged bees. YCPC supplementation significantly elevated total soluble protein content in newly emerged and nurse bees. Lower lipid levels were observed in newly emerged and nurse bees from the supplemented group and higher lipid reserves in were observed in supplemented foragers. No significant variation was observed in total carbohydrate content, except for a significant reduction in supplemented newly emerged bees. Acetyl cholinesterase activity was higher in younger bees and lower in foragers. A slight elevation in *α* and *β*-esterase expression was detected in the supplemented group, supporting enhanced detoxification capacity. YCPC enhances the biochemical capacity of honey bees, particularly in the early adult stages. However, glandular and morphological development were not affected.

## Simple Summary

The nutrition of honey bees is critical to colony survival and health. Honey bees are subjected to many factors that influence their survival, productivity, and role in plant pollination, particularly nutrition. Feeding honey bee colonies is important, especially during the fall and winter. Beekeepers feed their colonies protein and carbohydrate sources to meet the nutritional requirements of honey bees. However, the effects of these diets have not been fully studied. The current study evaluated the effects of a protein-enriched supplementary diet on the morphometric and biochemical characteristics of worker honey bees at different ages compared with control colonies. The supplemented diet affected the content of biochemical parameters, particularly total soluble protein, in younger worker honey bees. Colonies receiving the supplemented diet also showed slightly higher mean values of hypopharyngeal gland (HPG) measurements; however, these differences were not statistically significant. Overall, the results suggest that the supplementary diet mainly affected biochemical traits rather than morphological development.

## 1. Introduction

Beekeepers often face colony mortality rates of up to 40% annually. The recent massive losses of honey bee colonies worldwide have led to extensive research efforts to identify the underlying causes. Many factors have been identified, acting alone or in combination, including pathogens, malnutrition, and pesticides [1]. In addition, climate change, loss of plant biodiversity, and increased exposure to environmental stressors further contribute to colony decline. Among these factors, nutrition and plant sources play a major role in maintaining colony health [2].

Pollen nutrition is essential for the health of honey bees, as it helps bees resist stress from food scarcity and diseases such as Nosema and Varroa mites, and reduces their sensitivity to pesticides. Moreover, the diversity of plant sources of pollen makes bees more resistant to stress by enhancing the activities of enzymes associated with immunity [3,4]. Therefore, adequate and diverse nutrition is a key factor in maintaining healthy colonies, particularly under stressful environmental conditions [5].

The use of artificial diets has become an important management strategy during periods of pollen scarcity. The correct artificial diet can improve the nutritional balance and well-being of the colony. The importance of pollen in the diet should not be underestimated; planting appropriate plant species can increase the nutritional quality of a colony [6,7].

Beekeepers often do not have much choice during periods of pollen scarcity and must feed their colonies with pollen substitutes or regular sugar to produce stronger and healthier colonies [8,9]. Feeding colonies with an insufficient amount of natural pollen or inferior pollen substitutes may lead to losses of bee colonies. Thus, protein supplementation represents a critical management tool to support colony health during dearth periods when natural pollen is limited [10].

The development of hypopharyngeal glands (HPGs) varies with diet and is strongly related to protein intake in pollen-based diets. The acinus surface area of the HPG differs depending on the diet. The largest area was recorded when honey bee workers were fed bee bread, followed by a mixture of yeast, gluten, sugar, and pollen [11].

In addition to morphological traits, protein-rich diets are essential for maintaining physiological balance in honey bee workers. Nutritional protein intake has been linked to increased levels of abdominal lipid content and HPG development [12] and improved carbohydrate metabolism to support energy demands [13]. Dietary protein supplementation may also help restore acetylcholinesterase (AChE) activity in honey bees exposed to oxidative or neurotoxic stress [14]. These morphological and biochemical responses can vary with worker age, reflecting differences in physiological roles within the colony.

Based on these considerations, the present study aimed to evaluate the effects of supplementary feeding (the YCPC diet) on both morphological (HPGs, wax glands, and stinging apparatus) and biochemical parameters (i.e., total proteins, carbohydrates, lipids, and AChE activity) in worker honey bees at different developmental stages (i.e., newly emerged, nurse, and forager), in comparison with the control, as a part of ongoing projects on honey bees and bee products [1,15,16].

## 2. Materials and Methods

### 2.1. Study Area

This work was carried out in the apiaries of the Plant Protection Research Institute at El-Qanater El Khayreya, Qaluobia Governorate. El-Quanater El Khayreya, located at Latitude: 30°11035.77″ N and Longitude: 31°08′13.31″ E (https://latitude.to/map/eg/egypt/cities/al-qanatir-al-khayriyah (accessed on 25 June 2026)), is an agricultural region rich in diverse plant species, including crops and medicinal and aromatic plants. This floral diversity provides continuous forage that supports honey bees’ survival year-round.

### 2.2. Experimental Design

The experiment was conducted during the autumn and winter seasons. Six honey bee colonies headed by open-mated local Carnica queens and having relatively similar strengths (5–6 combs covered with adult bees) were used.

Colonies were divided into two groups; each group comprised three colonies (replicates). The first group (apiary I, supplemented group) received a supplementary protein and carbohydrate-enriched diet. The second group (apiary II, control group) did not receive any supplementary diet. The two apiaries were in the same geographical region (about 5 km apart) and were maintained under comparable environmental conditions. In addition, the agricultural practices and seasonal flowering periods were the same.

The supplemented diet contained Brewer’s yeast and chickpea cake fortified with bee pollen as a protein supplement (YCPC). The ingredient ratio was 2:3:3:1:15 for dried brewer’s yeast, chickpea meal, honey, pollen, and sugar, respectively. The chemical composition of the supplemented diet is shown in Table S1 [17]. In addition, the supplemented colonies were treated with formic acid during the experiment for Varroa mite control. Supplemented colonies received 50 g/colony/week for 6 months during the autumn and winter seasons [17].

Honey bee (newly emerged, nurse, and forager bees; n = 3 bees/age/colony/group) were collected at the end of winter. The honey bee samples were collected based on the method described by [18]. Using queen caging, newly emerged bees (1 day old) were obtained. Nurse bees were collected from the brood area of the comb. Forager bees were collected from the hive entrance or landing board.

### 2.3. Morphometric Analysis of Honey Bee Glands

The HPGs, the second wax gland mirror, and the stinging apparatus were dissected and measured.

#### 2.3.1. Hypopharyngeal Glands (HPGs)

At the end of winter, newly emerged, nurse, and forager bees (n = 3 bees/age/colony) were collected for dissection and gland examination. The width, length, and number of HPG acini were measured using a light microscope at 100× magnification with a micrometric lens and a micrometric slide. The micrometric lens was calibrated using the micrometric slide at 100× magnification (Figure S1) [19].

#### 2.3.2. Wax Gland

Worker honey bees, including nurse and forager bees (n = 3 bees/age/colony), were collected and dissected for wax gland mirror examination. The mean longitudinal and transverse dimensions of the wax mirror (plate) of the second wax gland on the fifth sternite were measured using a micrometric lens under a light microscope at 40 × magnification. The micrometric lens was calibrated with a micrometric slide at the same magnification (Figure S2) [20].

#### 2.3.3. Stinging Apparatus

Newly emerged, nurse, and forager bees (n = 3 bees/age/colony) were collected and dissected. The head was removed from the anterior end, and the sting apparatus was pulled from the posterior end and separated from the digestive canal. The length of the sting apparatus and the width of the poison sac were measured using a micrometric lens under a light microscope at 40× magnification. The micrometric lens was calibrated with a micrometric slide at 40× magnification (Figure S3) [21].

### 2.4. Biochemical Analysis

Newly emerged, nurse, and forager bees (n = 3 bees/age/colony) were sampled after receiving the supplemented diet, then weighed, and frozen for biochemical assays. The frozen samples were homogenized in distilled water using a Teflon homogenizer surrounded by a jacket of crushed ice, then centrifuged at 4000 rpm for 10 min. The supernatants were used for the estimation of total soluble proteins and AChE activity estimation, whereas the body homogenates were used for total proteins, carbohydrates, lipids, and AChE analyses. All chemicals and reagents (analytical grade) were used in the study were from Sigma Chemicals Company (London, UK).

#### 2.4.1. Total Soluble Proteins

The total soluble protein content of worker honey bees (n = 3/age/group) was estimated calorimetrically, as described by Gornall et al., using the Biuret reagent. Five milliliters of the reagent were added to 0.2 mL of the honey bees’ homogenate and incubated at 20–25 °C for 30 min. The absorbance of the sample was then measured against a Biuret reagent blank at 546 nm [22].

#### 2.4.2. Total Carbohydrates

The total carbohydrate content was estimated using the method described by Crompton and Birt. Worker honey bees were homogenized in 0.3 N HCIO_4_ (5 mL) at 0 °C for 1 min. The homogenate was kept on ice for a further 10 min. Insoluble matter was removed by centrifugation for 3 min at 2000 rpm. Then, 100 mL of the acid extract was added to a colorimetric tube containing 0.5 mL of phenol (20%). Then, 5 mL of concentrated sulfuric acid was added rapidly with shaking. The tubes were allowed to stand for 10 min, and then they were shaken and placed in a water bath at 25–30 °C for 10–20 min before measurement. Blanks were prepared by substituting distilled water for the sugar solution. The absorbance of the characteristic yellow–orange color was measured at 490 nm against a blank. The absorbance was measured colorimetrically at 490 nm [23].

#### 2.4.3. Total Lipids

The total lipid content of worker honey bees (n = 3/age/group) from the supplemented and control groups was measured according to the method of Zollner and Kirsch, using kits from Diamond Diagnostics. Lipids react with sulfuric and phosphoric acid, and vanillin to form a pink-coloured complex, which is estimated colorimetrically at 545 nm [24].

#### 2.4.4. Acetyl Cholinesterase (AChE)

The activity of AChE was estimated by the method of Simpson et al. The assay utilized 200 µL of enzyme solution, 0.5 mL of phosphate buffer (0.067 M, pH 7), and 0.5 mL of AchBr (3 mM) substrate solution [25].

### 2.5. Native Electrophoretic Isoenzyme Assay

Native electrophoretic patterns of known weights (0.2 g) of bee tissues were obtained by homogenizing them in the extraction buffer (1 mL) and then centrifuging them for 5 min at 10,000 rpm. From each group, equal volumes of the individual supernatants were mixed in one tube and used as one sample. The concentration of total protein was quantified in all pooled samples using the method described by Bradford (1976) [26]. All samples were diluted with loading dye to equalize protein concentration in all wells during the electrophoretic assays. Polyacrylamide gel electrophoresis (PAGE) was used for the separation of the native isoenzymes. The electrophoretic esterase (EST) patterns were assayed by incubating the native gel in conditioning buffer to optimize enzyme activity, then stained with a reaction mixture containing Fast Blue RR (as a dye coupler) along with *α*- and *β*-naphthyl acetate (as substrates) for detecting *α*- and *β*-EST, isoenzymes, respectively [27]. The *α*-EST isoenzymes appeared as brown bands and the *β*-EST isoenzymes as pink bands.

### 2.6. Data Analysis

Statistical analyses were conducted using Statistical Package for the Social Sciences(SPSS) version 25 (International Business Machines Corporation (IBM), New York, NY, USA). To evaluate the influence of worker age, the supplemented diet, and their interaction, a two–way ANOVA was carried out. Each parameter was tested in triplicate, and when significant variation was observed, Tukey’s HSD test was employed for multiple comparisons. Data are expressed as mean values with their standard deviations (SD), and differences were considered significant *p* < 0.05.

After photographing the PAGE plates, the Quantity One software (Version 4.6.2) was used to analyze relative mobility (Rf), quantity (Qty), and percentage (B%) of the electrophoretically separated bands. Electrophoretic banding patterns were compared based on the presence or absence of isoenzyme bands, relative mobility (Rf), and band intensity percentage (B%) among the tested groups.

## 3. Results

### 3.1. Morphometric Analysis of Honey Bee Glands

#### 3.1.1. The Hypopharyngeal Gland

**Figure 1:**
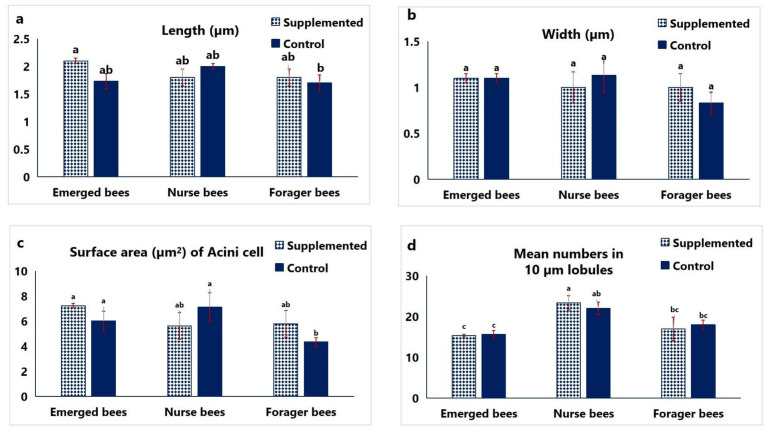
Morphometric parameters of acini cells in hypopharyngeal gland lobules of worker honey bees at the end of winter. (**a**) length (µm), (**b**) width (µm), (**c**) surface area (µm^2^), and (**d**) number of acini cells per 10 µm gland lobules. Data represent mean ± SE of three independent replicates. Different letters indicate significant differences at *p* ≤ 0.05.

Data in Table S2 and Figure 1 show the effects of worker age and supplementary diet on HPG acini. Acini length, width, and surface area showed no significant differences due to age, supplementation, or their interaction, although slight variations were observed among groups (Figure 1a–c). The number of acini per 10 µm lobule was significantly affected by age, being highest in nurse bees and lowest in newly emerged bees, while foragers showed intermediate values (Figure 1d). Supplementation had no significant effect on acini number; however, the interaction between age and supplementation was significant. Therefore, among the HPG traits examined, only acini number showed a significant age-related response.

#### 3.1.2. The Wax Gland

**Figure 2:**
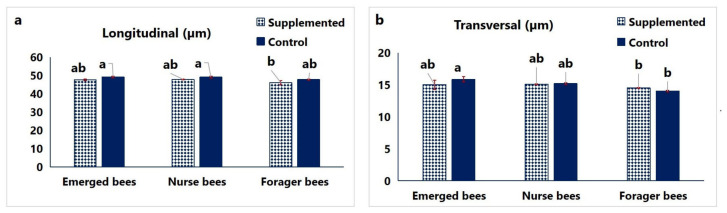
Mean dimensions of the second wax gland mirror in worker honey bees. (**a**) longitudinal (µm) measurements, (**b**) transversal (µm) measurements. Data represent mean ± SE of three independent replicates. Different letters indicate significant differences at *p* ≤ 0.05.

Data in Table S3 and Figure 2 show the longitudinal and transverse dimensions (µm) of the second wax gland mirror in worker honey bees at the end of the winter. The longitudinal dimension was significantly higher in the control colonies than in the supplemented colonies (Table S3; Figure 2a). Regarding age, forager bees had significantly lower values than newly emerged and nurse bees. For the transverse dimension, supplementation had no significant effect, whereas age significantly affected the measurements, with forager bees showing the lowest values compared with newly emerged and nurse bees (Figure 2b). The significant interaction between supplementation and age was detected for both dimensions.

#### 3.1.3. Stinging Apparatus and Poison Sac

**Figure 3:**
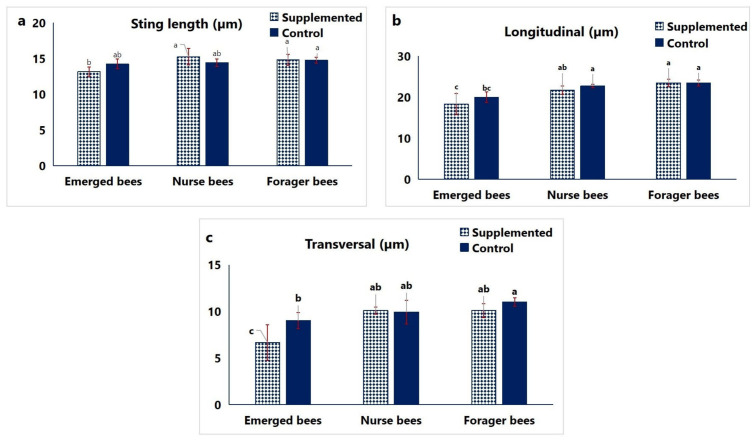
Morphometric measurements of the stinging apparatus in worker honey bees at the end of winter. (**a**) mean sting length (µm), (**b**) mean longitudinal dimensions of poison sac (µm), (**c**) mean transverse dimensions (µm) of poison sac. Data represent mean ± SE of three independent replicates. Different letters indicate significant differences at *p* ≤ 0.05.

The data in Table S4 and Figure 3 show the sting length and poison sac dimensions of worker honey bees at the end of winter. Supplementation had no significant effect on any trait, as the overall means in supplemented and control colonies were similar. Age significantly affected all traits; sting length was the shortest in newly emerged bees (13.71 ± 0.33 µm) and the longest in nurse bees (14.83 ± 0.37 µm) (Figure 3a). The supplemented diet did not show a significant difference in the longitudinal and transverse poison sac dimensions (Figure 3b,c). Age had a significant effect on the longitudinal and transverse poison sac dimensions of worker honey bees. No significant interaction between the supplemented group and age was detected for sting length or longitudinal poison sac dimension; however, a significant interaction between age and diet was observed for the transverse poison sac dimensions.

### 3.2. Biochemical Analysis

#### 3.2.1. Total Soluble Proteins

**Figure 4:**
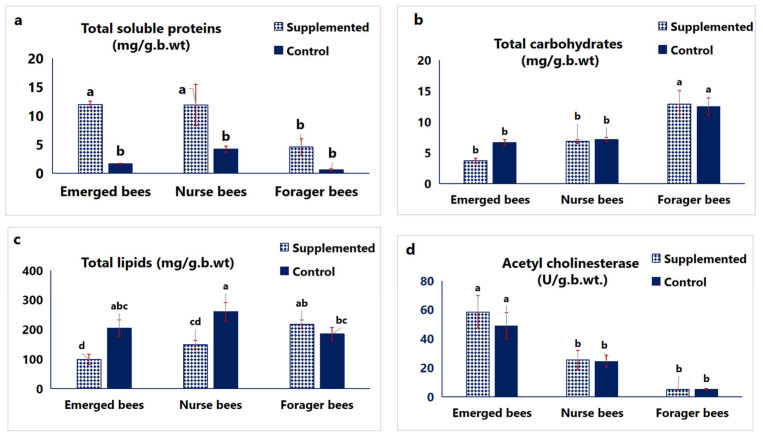
Biochemical parameters in homogenates of newly emerged, nurse, and forager honey bee workers at the end of winter in supplemented and control groups. (**a**) total soluble proteins, (**b**) total carbohydrates, (**c**) total lipids, and (**d**) acetyl cholinesterase (AChE). Data represent mean ± SE of three independent replicates. Different letters indicate significant differences at *p* ≤ 0.05.

The total soluble protein levels measured in the bodies of newly emerged, nurse, and forager worker honey bees are shown in Table S5 and Figure 4. Protein content was significantly affected by age, supplementation, and their interaction. Newly emerged and nurse bees in the supplemented group recorded the highest protein levels (11.98 ± 0.59 and 11.86 ± 3.596 mg/gb.wt), respectively, which were significantly higher than those in the control group (1.6 ± 0.07 and 4.20 ± 0.53 mg/gb.wt), respectively. Forager bees in supplemented colonies also exhibited lower protein content (4.55 ± 1.48 mg/gb.wt) compared with that in control colonies (0.6 ± 0.20 mg/gb.wt). 

#### 3.2.2. Total Carbohydrates

No significant differences in the total carbohydrate content were observed between the supplemented and control groups. Total carbohydrate content varied noticeably among the worker ages (Table S5 and Figure 4), with the lowest values recorded in newly emerged bees and the highest in forager bees. The interaction between supplementation and age was significant; the newly emerged bees from the supplemented group had the lowest carbohydrate level (3.70 ± 0.410 mg/g.b.wt), while the forager bees had the highest value. Overall, carbohydrate content increased with worker age, while supplementation had no significant effect.

#### 3.2.3. Total Lipids

The total lipid content in newly emerged, nurse, and forager worker bees reared in supplemented and control colonies is shown in Table S5 and Figure 4. Lipid levels varied with both age and dietary supplementation. Age and the supplemented diet, as well as their interaction, significantly affected the lipid content. Newly emerged and nurse bees from the supplemented group showed lower lipid levels compared with those from the control group. Nurse bees exhibited higher lipid content in control colonies than in supplemented colonies.

##### Acetyl Cholinesterase

The AChE activity measured in newly emerged, nurse, and forager bee workers from the supplemented and control colonies is shown in Table S5 and Figure 4. Overall, AChE activity did not differ significantly between the supplemented and control groups. Age had a pronounced effect on AChE activity, with newly emerged workers recording the highest values (53.53 ± 6.73 U/gb.wt), while forager bees displayed the lowest enzyme activity (4.99 ± 0.23 U/gb.wt). This pattern indicates that AChE activity declines sharply with the worker’s age. The interaction between age and supplementation was significant. The newly emerged showed the highest value (58.31 ± 11.08 U/gb.w) in the supplemented group.

### 3.3. Electrophoretic α-Esterase and β-Esterase Analysis

**Figure 5:**
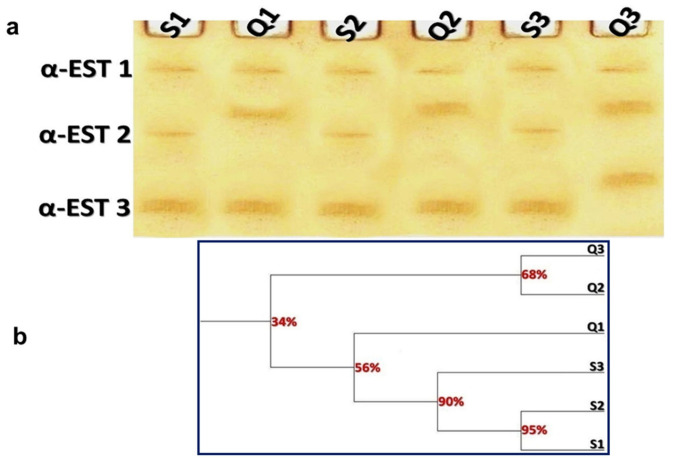
(**a**) Native electrophoretic patterns of α-esterase (*α*-EST) isoenzymes show differences in the physiological state among different honey bee groups; (**b**) phylogenetic tree of electrophoretic *α*-EST isoenzyme. Each lane in the supplemented groups (Q) was compared with the corresponding control groups(S). Q1, Q2, and Q3: Newly emerged, nurse and forager bees from the supplemented group, respectively. S1, S2, and S3: Newly emerged, nurse and forager bees from the control group, respectively.

The electrophoretic analysis of α-esterase (*α*-EST) isoenzymes exhibited characteristic banding patterns among different worker honey bees groups (Table 1, Figure 5 and Figure S4). Analysis of relative mobility (Rf) and band percentage (B%) revealed clear differences between supplemented and control groups (Table 1). For *α*-EST 1, two bands were detected: the main band (Rf 0.16–0.17) showed the highest intensity in nurse bees of the supplemented group (41.16%), followed by newly emerged bees (39.85%), and forager bees (38.94%), whereas control foragers exhibited comparable activity (40.05%). An additional *α*-EST 1 band (Rf 0.34–0.39) appeared only in the supplemented worker bees with intensities ranging from 37.37% in newly emerged bees to 41.16% in nurses and 38.96% in foragers. Regarding *α*-EST 2, it was absent in newly emerged bees from the supplemented group, while it was present in the control (Rf 0.49, B% 29.46%). Another *α*-EST 2 band (Rf 0.73, B% 22.10) was uniquely detected in foragers from the supplemented group.

In contrast, *α*-EST 3 appeared in all groups, with the highest expression in newly emerged bees (35.61% in the control group vs. 22.78% in the supplemented group), while foragers in both groups had lower levels, reflecting an age-related decline in *α*-esterase activity.

Dendrogram analysis (Figure 5 and Figure S4) revealed differences in *α*-EST isoenzymatic expression patterns between supplemented and control groups. High isoenzymatic expression similarity was observed among control workers, ranging from 90 to 95%, whereas lower similarity values were detected among supplemented workers, particularly between nurse and forager bees (68%). The lowest similarity value (34%) was observed between supplemented and control bees, reflecting pronounced variation in *α*-EST expression patterns associated with dietary supplementation. 

**Figure 6:**
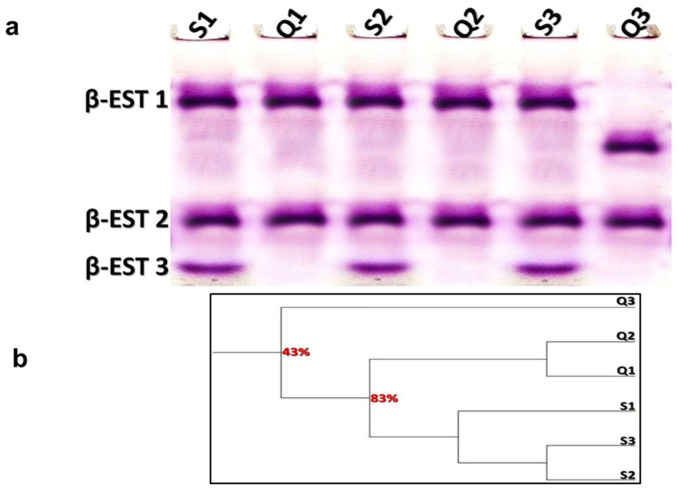
(**a**) Native electrophoretic patterns of *β*-esterase (β-EST) isoenzymes show differences in the physiological state among the differently treated honey bee groups; (**b**) phylogenetic tree of electrophoretic *β*-esterase (*β*-EST) isoenzyme. Each lane of the treated groups (Q) is compared with the corresponding control groups (S). Q1, Q2, and Q3: Newly emerged, nurse and forager bees for the supplemented group, respectively. S1, S2, and S3: Newly emerged, nurse, and forager bees for the control group, respectively.

The electrophoretic profiles of *β*-esterase (*β*-EST) isoenzymes also revealed marked variability among the tested worker honey bee groups (Table 2, Figure 6 and Figure S5). For *β*-EST 1, the highest B% values were observed in the supplemented groups, with 50.53% in newly emerged bees and 50.78% in nurse bees, whereas in the control group, the highest activity was detected in forager bees (39.08%). An additional band of *β*-EST 1(RF = 0.43, B% 50.10) appeared exclusively in supplemented foragers. Regarding *β*-EST 2, this isoenzyme was consistently expressed across all groups, with slightly higher intensities in the supplemented bees. By contrast, *β*-EST 3 was absent in all supplemented groups but present in controls, with intensities ranging from 22.07% in nurse bees to 23.62% in newly emerged bees, reflecting a supplemented-related disappearance of this isoenzyme.

The dendrogram analysis (Figure 6 and Figure S5) documented high isoenzymatic expression similarity (83%) among the control bees (newly emerged, nurse, and foragers). In contrast, supplemented foragers (Q3) clustered separately and showed lower similarity values compared with the other groups, indicating marked variation in *β*-EST expression patterns associated with dietary supplementation.

### 3.4. Discussion

HPGs play an important role in the life of worker honey bees, especially nurse bees, due to their role in royal jelly secretion, which is essential for brood nourishment and the queen’s diet. Their development is strongly dependent on protein intake, particularly from pollen, which supports acinar growth and glandular activity [28]. A diet rich in pollen is therefore essential for the normal development and function of HPGs, with secretion levels positively correlated with glandular activity and acini size.

Pollen feeding has been shown to enhance acini size and secretory activity, whereas feeding on sucrose alone results in reduced gland development [29]. In the present study, colonies supplemented with YCPC recorded slightly higher mean values of acini length, width, and surface area, and acini number, compared to the control group. However, these differences were not statistically significant, indicating that the supplementation level was insufficient to induce measurable structural changes in HPGs under the conditions of this experiment. This finding agrees with that of Al-Ghamdi et al., who reported that maximum development and larger acini surface area were observed in bees fed bee bread, followed by pollen loads, and a mixture of yeast, gluten, and sugar (1:1:2). Similarly, De Grandi-Hoffman et al. observed no significant difference in HPG volume between workers consuming higher pollen levels and those receiving protein. However, bees fed only sugar syrup had lower protein concentrations and smaller HPGscompared to the protein-fed groups [30]. Together, these results suggest that HPG development is maintained within a functional range when baseline nutritional requirements are met, with only subtle variation in response to supplementation.

Additionally, wax production is a key function of worker honey bees, with wax secreted from paired, smooth, oblong areas called wax mirrors, and wax production is influenced by age, nutrition, and colony demand. Increased secretory activity causes wax gland cells to elongate and become more slender [31]. In the present study, the longitudinal dimension of the second wax mirror differed significantly between supplemented and control groups, whereas the transverse dimension was not affected, indicating a limited effect of the supplemented diet. In contrast, age had a clearer influence, with more developed glands in nurse bees than in foragers. These findings are in partial agreement with Shawer and Mousa, who indicated that wax glands are not affected by diet type or worker age, although wax secretion may be affected [32]. Similarly, seasonal variation in gland development has been reported, with higher variation in gland development has been reported, with wax glands more developed in nurses than in foragers [33]. Overall, the present results suggest that wax gland development is influenced more by worker age and physiological role than by dietary supplementation.

The stinging apparatus is a key defensive structure in bee workers, with venom production increasing during early adult life and peaking at the onset of foraging and defensive activities [34]. In the present study, sting length and poison sac dimensions varied significantly with age, with newly emerged bees showing the smallest values and forager bees the largest. In contrast, the supplemented diet had no significant effect on these traits, indicating that the development of the defensive system is largely independent of dietary supplementation under the tested conditions. Previous studies have shown that dietary supplementation can enhance the volume of the acid gland sac and venom production in honey bee workers [35,36]. Such physiological effects do not necessarily translate into measurable changes in sting morphology under the present experimental conditions.

The limited morphological response observed in HPGs, the stinging apparatus, and the wax gland was accompanied by changes in key biochemical parameters, indicating that dietary supplementation primarily influenced physiological rather than morphological traits. These biochemical changes, including changes in total proteins, carbohydrates, lipids, and AChEwhich are essential for royal jelly production, venom synthesis, energy metabolism, and neuromuscular function, were influenced by the type of supplemental feeding provided to the colonies [37,38,39].

Bee pollen and chickpeas in the YCPC diet offer a broad spectrum of ingredients, providing high nutritional value that is beneficial for regulating the functions of several internal organs. This is consistent with earlier findings, which indicate that pollen contains diverse nutrients, including proteins, lipids, amino acids, vitamins, and minerals, all of which play a vital role in growth and development [40]. Similarly, chickpea is a nutritious legume, rich in protein, carbohydrates, dietary fiber, essential vitamins, and minerals [41]. This nutritional composition likely contributed to the observed changes in biochemical parameters, particularly in younger workers.

However, the YCPC diet consisted of multiple nutritional components (i.e., proteins and carbohydrates). The influence of the diet on honey bee responses (i.e., biochemical and morphological changes) should be interpreted as a whole rather than as the individual contribution of a specific ingredient. Further studies evaluating the dietary ingredients separately would help clarify their relative contributions to honey bee physiological responses.

Our findings demonstrated that total soluble protein levels were significantly higher in newly emerged and nurse workers from supplemented colonies, while forager bees showed no significant response. This indicates that the effect of the supplemented diet is age-dependent and more pronounced during early developmental stages. The higher protein content in young workers following dietary protein intake likely reflects enhanced synthesis pathways supporting brood care and nourishment [42]. The YCPC supplement provides essential amino acids and high-quality protein, which are critical for adult bee nutrition, brood development, and glandular function [43]. Moreover, adequate protein intake is also known to support honey bee immune function [37,44,45] and to influence cognitive abilities such as learning and memory throughout development [46]. This result is consistent with the limited structural changes observed in HPGs, suggesting that biochemical responses may precede detectable morphological variation.

Carbohydrates are a vital source of energy, supplying the required fuel for foraging activity and sustaining internal colony function [47]. In the current study, total carbohydrate content did not differ significantly between supplemented and control bees within the same age group, indicating that supplementation had minimal influence on carbohydrate reserves. However, carbohydrate levels increased with age, reaching the highest levels in foragers, which is consistent with their elevated energetic demands during sustained flight [48]. This pattern confirms that carbohydrate dynamics are primarily associated with age-related behavioral roles rather than dietary supplementation.

Regarding total lipids, lipid reserves varied significantly with worker age and differed between the supplemented and control groups. Newly emerged and nurse bees from YCPC-supplemented colonies exhibited lower lipid levels than controls, whereas foragers in supplemented colonies showed higher lipid content. These findings suggest that the dietary supplementation may influence lipid allocation differently across worker stages. The lower levels observed in newly emerged and nurse bees in supplemented colonies may be associated with increased metabolic activity or utilization of reserves; on the other hand, the absence of protein and sugar supplementation in control colonies may have reduced brood rearing due to nutritional deficiency, thereby lowering energy expenditure and maintaining lipid reserves in young workers. In contrast, malnutrition may have forced early foraging, leading to energy exhaustion, explaining the lower lipid content observed in foragers [49]. Additionally, the formic acid treatment may induce physiological stress, resulting in higher energy consumption [50]. Both age and supplementation significantly affected lipid content, and a significant interaction between these factors was detected, indicating that the effect of supplementation differed among worker age groups. Overall, these results indicate that lipid metabolism is shaped by both age-related physiology and dietary conditions.

AChE activity showed no significant difference between supplemented and control colonies within each age group, indicating that supplementation had minimal effect on enzyme dynamics. However, age exerted a strong influence, with newly emerged workers exhibiting the highest activity and foragers showing the lowest. This pattern is consistent with the functional role of AChE in neural signaling and behavioral maturation, where enzyme activity is linked to developmental transitions and task performance [51]. The reduced enzyme activity in foragers may result from direct contact with xenobiotic pollutants during foraging [52]. AChE remains a crucial biomarker for evaluating the physiological health of honey bees [53], with activity influenced by pesticide exposure [54,55], landscape differences [52], seasonal variations [56], bee caste, and brood rearing status [57]. These findings support the use of AChE as an indicator of age-related functional rather than dietary effects in the present study.

Similarly, esterases are essential enzymes involved in detoxification, metabolism, and xenobiotic resistance, and adaptation in honey bees. Their expression can be influenced by dietary intake, including protein [58,59]. The results indicated that protein supplementation influenced esterase activity and isoenzymatic expression patterns in honey bees. *α*-EST 1 activity was notably higher in newly emerged bees (39.85%) and nurse bees (41.16%) in the supplemented colonies compared to their respective controls (34.94% and 32.18%), suggesting that a protein-rich diet enhances α-EST expression, consistent with [58], who found that increased dietary protein improves enzymatic activity and physiological stability. The absence of *α*-EST 2 in most supplemented groups suggests possible suppression of this isoenzyme due to protein supplementation, indicating a selective enzymatic response [44]. Moreover, the lowest isoenzymatic expression similarity observed between supplemented and control forager bees confirms that dietary supplementation significantly alters enzyme expression, supporting the role of protein in regulating metabolic performance and enhancing stress adaptation. *β*-EST 1 expression was highest in supplemented groups, indicating enhanced detoxification enzyme activity and potentially improved honey bee resistance to environmental stressors [48]. Notably, *β*-EST 3 was absent in all supplemented groups, but present in all control worker groups, suggesting that dietary supplementation may shift reliance from *β*-EST 3 to alternative detoxification mechanisms, as shown by the elevated B% of *β*-EST 1 and *β*-EST 2 in supplemented groups.

## 4. Study Limitation

A limitation of the present study is that the supplemented and control colonies were maintained in separate apiaries, and only the supplemented colonies received formic acid treatment for *Varroa* control. Although both apiaries were located in the same geographical region and managed under comparable environmental conditions, these factors may have contributed to part of the observed variation among groups. In addition, the relatively small number of colonies and sampled workers should be considered when interpreting the findings. Therefore, further studies using identical management conditions are required to confirm the effects of dietary supplementation.

## 5. Conclusions

The nutrition of honey bees is critical to colony survival, particularly during the fall and winter seasons. Supplemental feeding with a diet containing Brewer’s yeast and chickpea cake fortified with bee pollen as a protein supplement positively affected selected biochemical parameters in worker bees (newly emerged, nurse, and forager), particularly by enhancing total soluble protein content in younger workers, while showing no significant effects on morphological traits such as hypopharyngeal gland development, second wax mirror size, stinger length, and venom sac dimensions. Supplemental feeding also influenced some physiological markers, including acetylcholinesterase activity and the expression of detoxification-related enzymes (*α*-EST and *β*-EST), which are essential for metabolism and physiological responses. The results reported in the current study should be considered preliminary findings on the effects of the supplementary diet on morphometric and biochemical parameters of honey bees under field conditions. However, these findings should be interpreted with caution because of the limitations of this study, and further studies with larger colony numbers and more controlled experimental conditions are warranted to confirm the effects of dietary supplementation.

## Figures and Tables

**Table 1:** α-esterase (α-EST) isoenzymes of supplemented and control honey bee workers at different ages.

α-EST Isoenzymes	Supplemented Group						Control Group					
	Newly Emerged Bees		Nurse Bees		Forager Bees		Newly Emerged Bees		Nurse Bees		Forager Bees	
	Rf	B%	Rf	B%	Rf	B%	Rf	B%	Rf	B%	Rf	B%
**α-EST 1**	0.17	39.85	0.17	36.57	0.16	38.94	0.16	34.94	0.17	32.18	0.16	40.0
	0.39	37.37	0.35	41.16	0.34	38.96	-	-	-	-	-	-
**α-EST 2**	-	-	-	-	-	-	0.49	29.46	-	-	0.47	34.8
	-	-	-	-	0.73	22.10	-	-	-	-	-	-
**α-EST 3**	0.88	22.78	0.88	22.27	-	-	0.87	35.61	0.88	33.12	0.88	25.1
**SI%**	66.67		66.67		33.33		-		-		-	

Rf: Relative mobility, B%: Band percent, and SI%: Similarity index.

**Table 2:** Electrophoretic *β*-EST isoenzymes of supplemented and control worker honey bees at different ages.

*β*-EST Isoenzymes	Supplemented Group						Control Group					
	Newly Bees Emerged Bees		Nurse Bees		Forager Bees		Newly Emerged Bees		Nurse Bees		Forager Bees	
	Rf	B%	Rf	B%	Rf	B%	Rf	B%	Rf	B%	Rf	B%
*β*-EST 1	0.255	50.53	0.26	50.78	-	-	0.25	35.75	0.25	38.34	0.26	39.08
	-	-	-	-	0.43	50.10	-	-	-	-	-	-
*β*-EST 2	0.72	49.47	0.73	49.22	0.73	49.90	0.73	40.62	0.72	39.59	0.73	38.63
*β*-EST 3	-	-	-	-	-	-	0.93	23.62	0.92	22.07	0.93	22.29
SI%	80.00		80.00		40.00		-		-		-	

Rf: Relative mobility, B%: Band percent, and SI%: Similarity index.

## Data Availability

The data that support the findings of this study are available upon reasonable request.

## Supplementary Materials

The following supporting information can be downloaded at: https://www.mdpi.com/article/10.3390/vetsci13070629/s1, Figure S1: (a) Dissection of the head of a worker bee for examination of the hypopharyngeal gland. (b): Lobule of the hypopharyngeal gland. (c) Calibration of a micrometric lens using a micrometric slide at 100× magnification. (d) Measurement of acini cell length in the hypopharyngeal gland. (e) Measurement of acini cell width in the hypopharyngeal gland. (f) Number of acini cells with a 10 μm length in the hypopharyngeal gland; Figure S2: (a) Dissection of the worker abdomen for wax mirror examination. (b) The sternite showing the wax mirrors of the wax gland. (c) Calibration of the micrometric lens using the micrometric slide at 40 × magnification. (d) Measurement of the length of the wax mirror (plate) of the second wax gland. (e) Measurement of the transverse width of the wax mirror (plate) of the second wax gland; Figure S3: (a) Stinging apparatus after separation from the digestive canal. (b) Measurement of the length of the stinging apparatus (c) Measurement of the longitudinal length of the poison sac. (d) Measurement of the width of the poison sac; Figure S4: (a) Electrophoretic α-esterase (α-EST) isoenzyme pattern 1. unprocessed image; 2. converted image; 3. unprocessed analyzed image; (b) Phylogenetic tree of electrophoretic α-esterase (*α*-EST) isoenzyme pattern (unprocessed image). Figure S5: (a) Electrophoretic *β*-esterase (*β*-EST) isoenzyme pattern 1. unprocessed image; 2. converted image; 3. unprocessed analyzed image; (b) Phylogenetic tree of electrophoretic *β*-esterase (*β*-EST) isoenzyme pattern (unprocessed image); Table S1: Chemical analysis of protein supplements was used in the current study; Table S2: Mophometric characteristics of hypopharyngeal gland (Length, width (µm), surface area, and number per 10 µm lobules in worker honey bees at the end of winter; Table S3: Longitudinal and transversal (µm) measurements of the second wax gland mirror of honey bee workers at the end of winter; Table S4: Morphometric measurements (µm) of the stinging apparatus and poison sac in honey bee worker at the end of winter; Table S5: Biochemical parameters in homogenates of newly emerged, nurse, and forager honey bee workers in supplemented and control groups at the end of winter.
