# Supplementary material for: Morphometric and Biochemical Parameters of Apis mellifera Workers Fed on Protein Supplement
Source: Vet Sci. 2026 Jun 27;13(7):629. doi: 10.3390/vetsci13070629 (PMC13431311; doi:10.3390/vetsci13070629)
Supplement: Supplementary file 1 [file vetsci-13-00629-s001.zip › vetsci-4364375-supplementary.pdf]

Supplementary Materials

Morphometric and Biochemical Parametersof *Apis mellifera* Workers Fed on Protein Supplement

Rasha S. Sakla[1], Aida A. Abd El-Wahed[1,*], Wael Mahmoud Aboulthana[2], Sobhia S. Sayed[1,*]

[1] Department of Bee Research, Plant Protection Research Institute, Agricultural Research Centre, Giza 12627, Egypt; rasha.s.sakla@gmail.com (R.S.S); aidaabd.elwahed@arc.sci.eg (A.A.A.E.-W), sobhiasaid@yahoo.com (S.S.S);

[2]Biochemistry Department, National Research Centre, 33 El Bohouth St. (Former ElTahrir St.), Dokki, Giza, P.O.12622, Egypt; wmkamel83@hotmail.com

*Corresponding author: aidaabd.elwahed@arc.sci.eg (A.A.A.E.-W), sobhiasaid@yahoo.com (S.S.S)

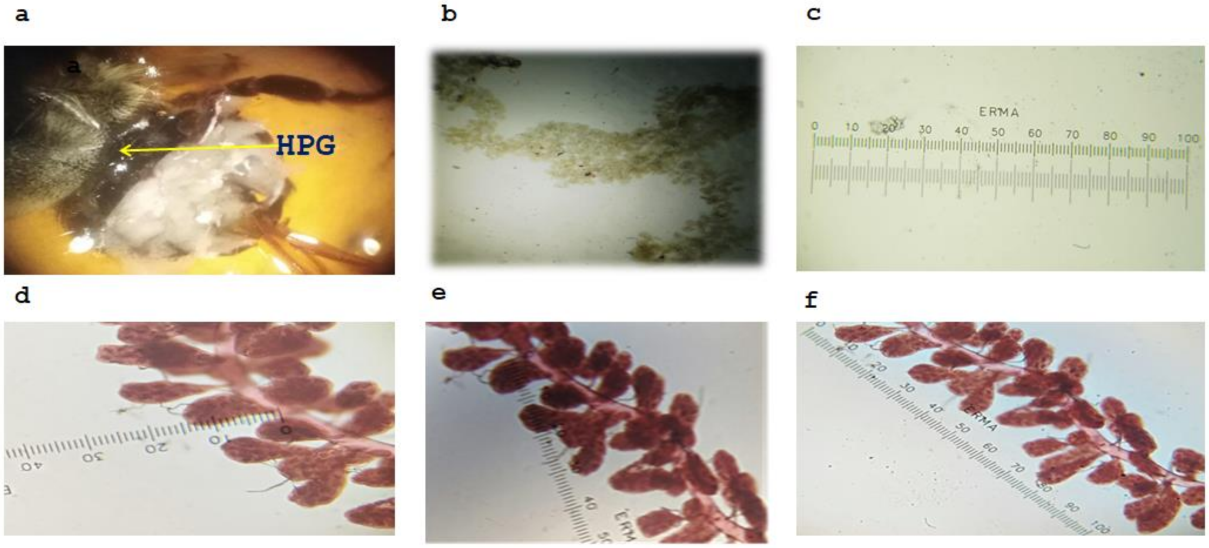

Figure S1: (a) Dissection of the head of a worker bee for examination of the hypopharyngeal gland. (b)**:** Lobule of the hypopharyngeal gland. (c) Calibration of a micrometric lens using a micrometric slide at 100× magnification. (d) Measurement of acini cell length of in the hypopharyngeal gland. (e) Measurement of acini cell width in the hypopharyngeal gland. (f) Number of acini cells with a10 μm length in the hypopharyngeal gland.

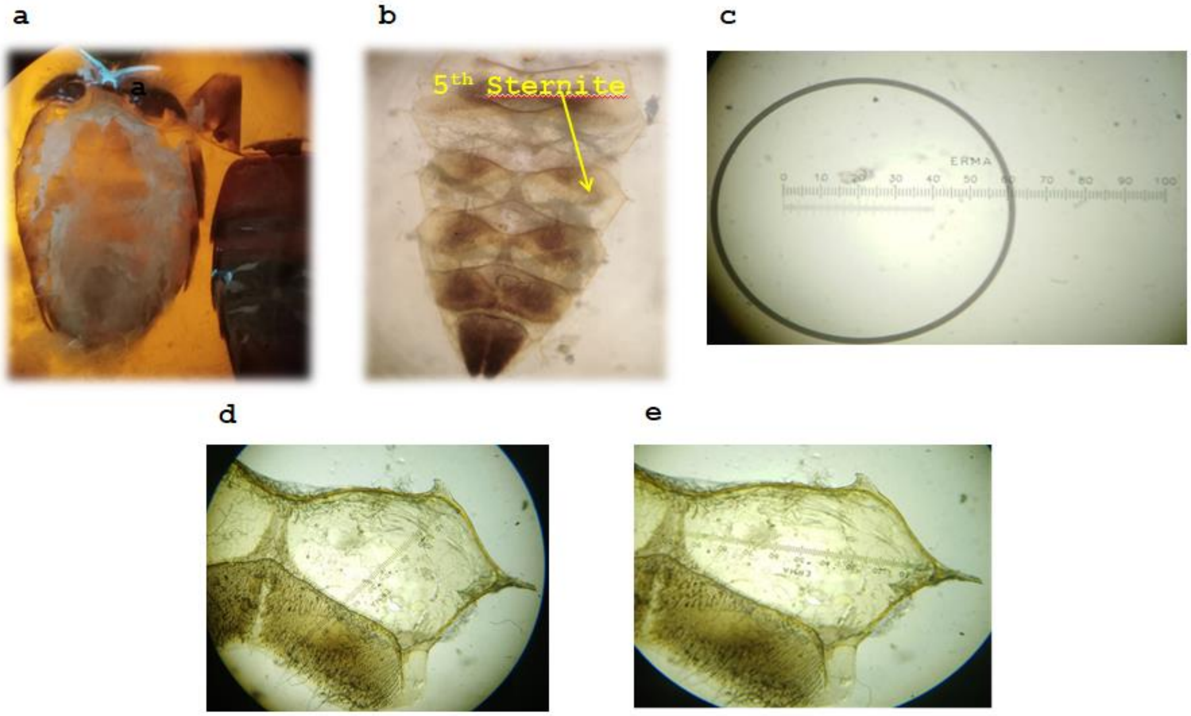

Figure S2: (a) Dissection of the worker abdomen for wax mirror examination. (b): The sternites showing the wax mirrors of the wax gland. (c) Calibration of the micrometric lens using a the micrometric slide at 40 × magnification. (d) Measurement of the length of the wax mirror (plate) of the second wax gland. (e) Measurement of the transverse width of the wax mirror (plate) of the second wax gland.

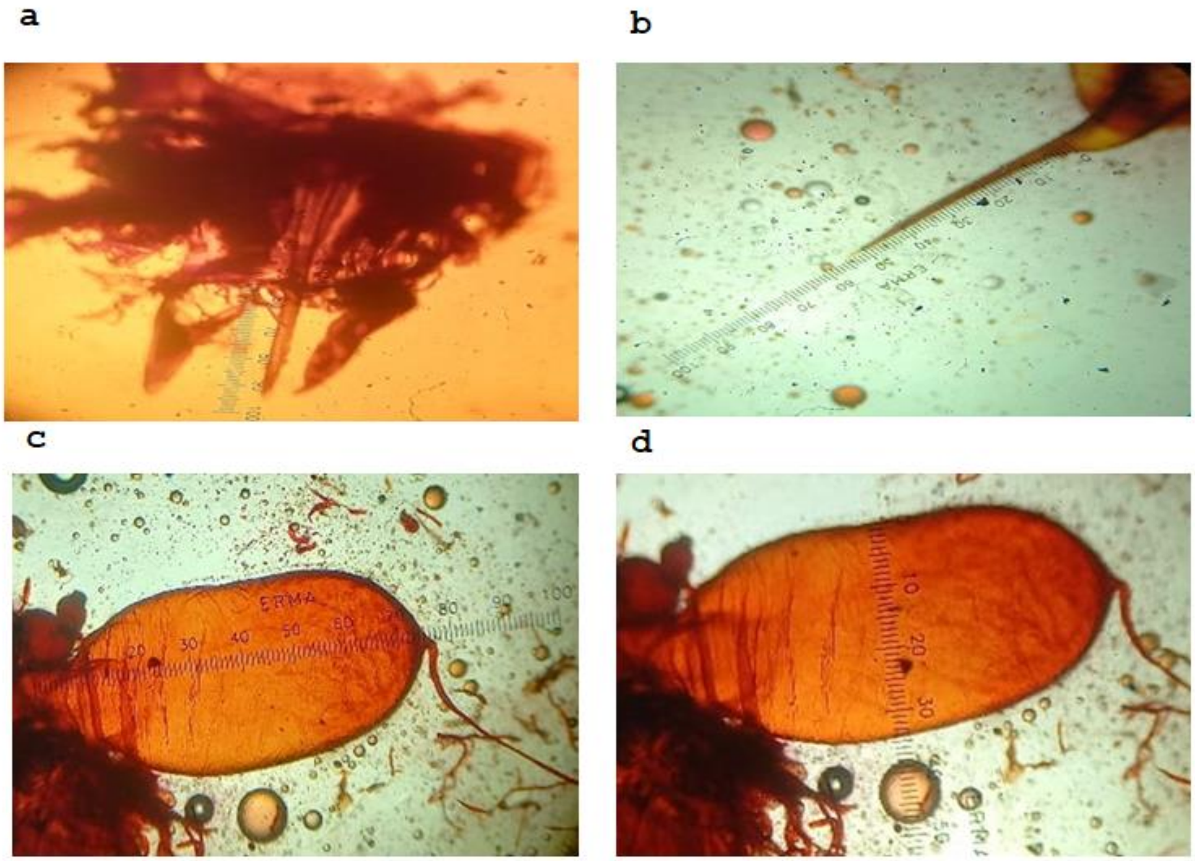

Figure S3: (a) Stinging apparatus after separation from the digestive canal. (b) Measurement of the length of the stinging apparatus (c) Measurement the longitudinal length of the poison sac. (d) Measurement of the width of the poison sac

1.

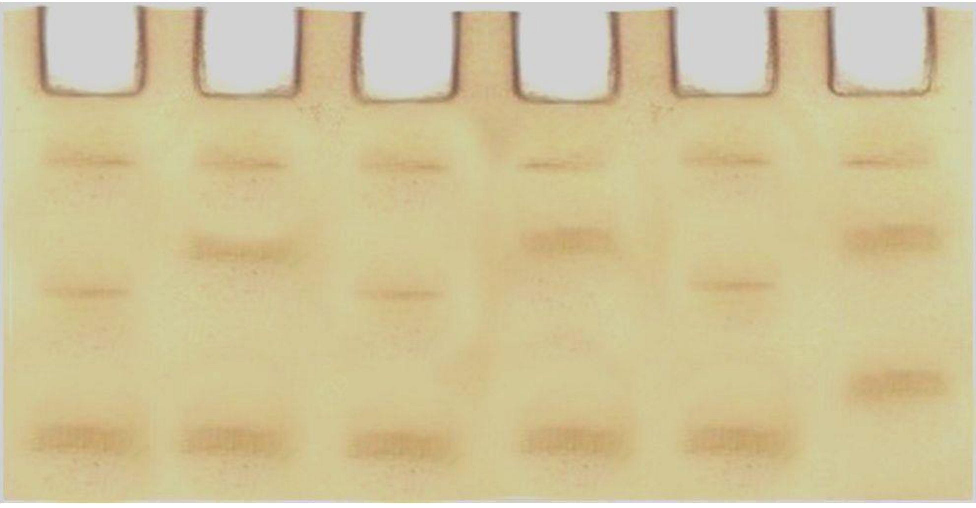

2.

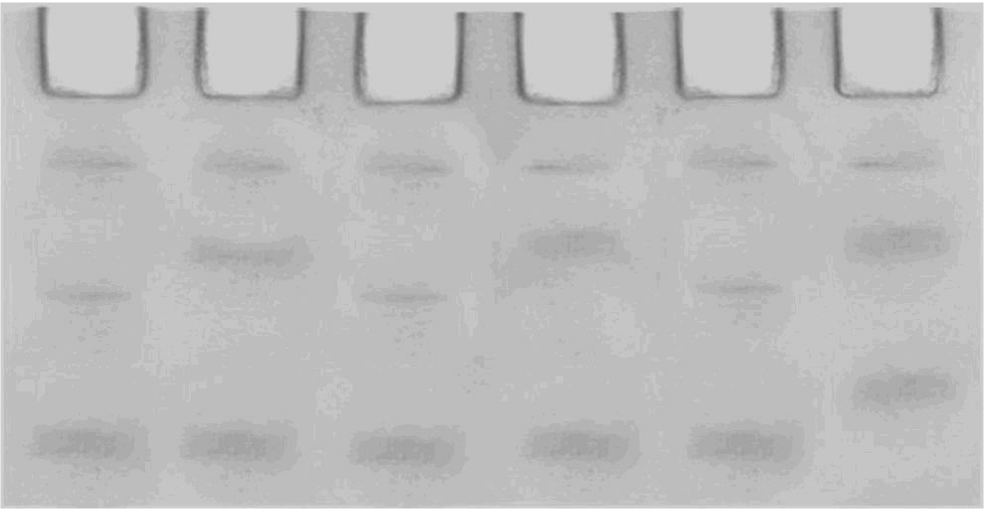

3.

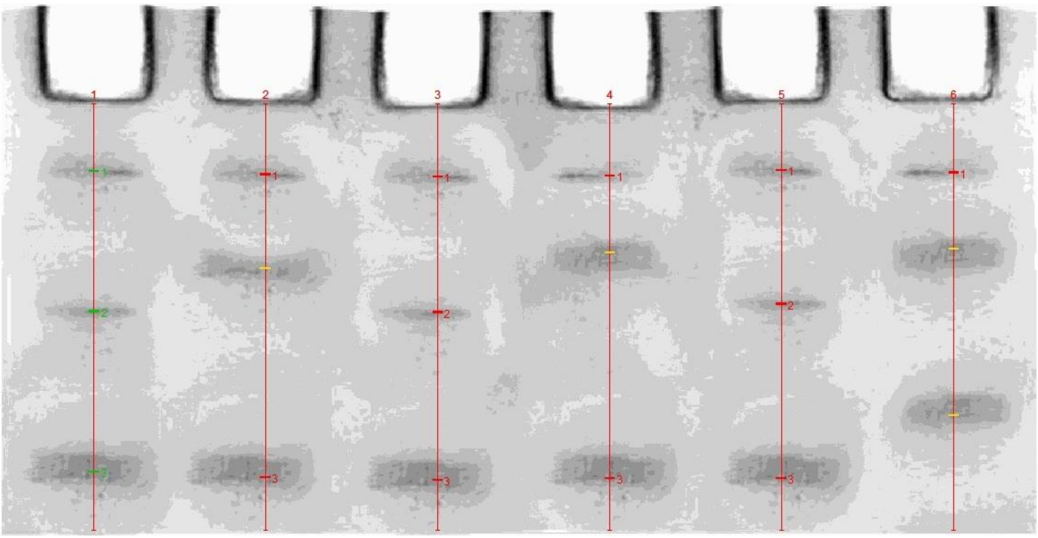

Figure S4. (a) Electrophoretic α-esterase (α-EST) isoenzyme

pattern 1. unprocessed image; 2. converted image; 3. unprocessed analyzed image.

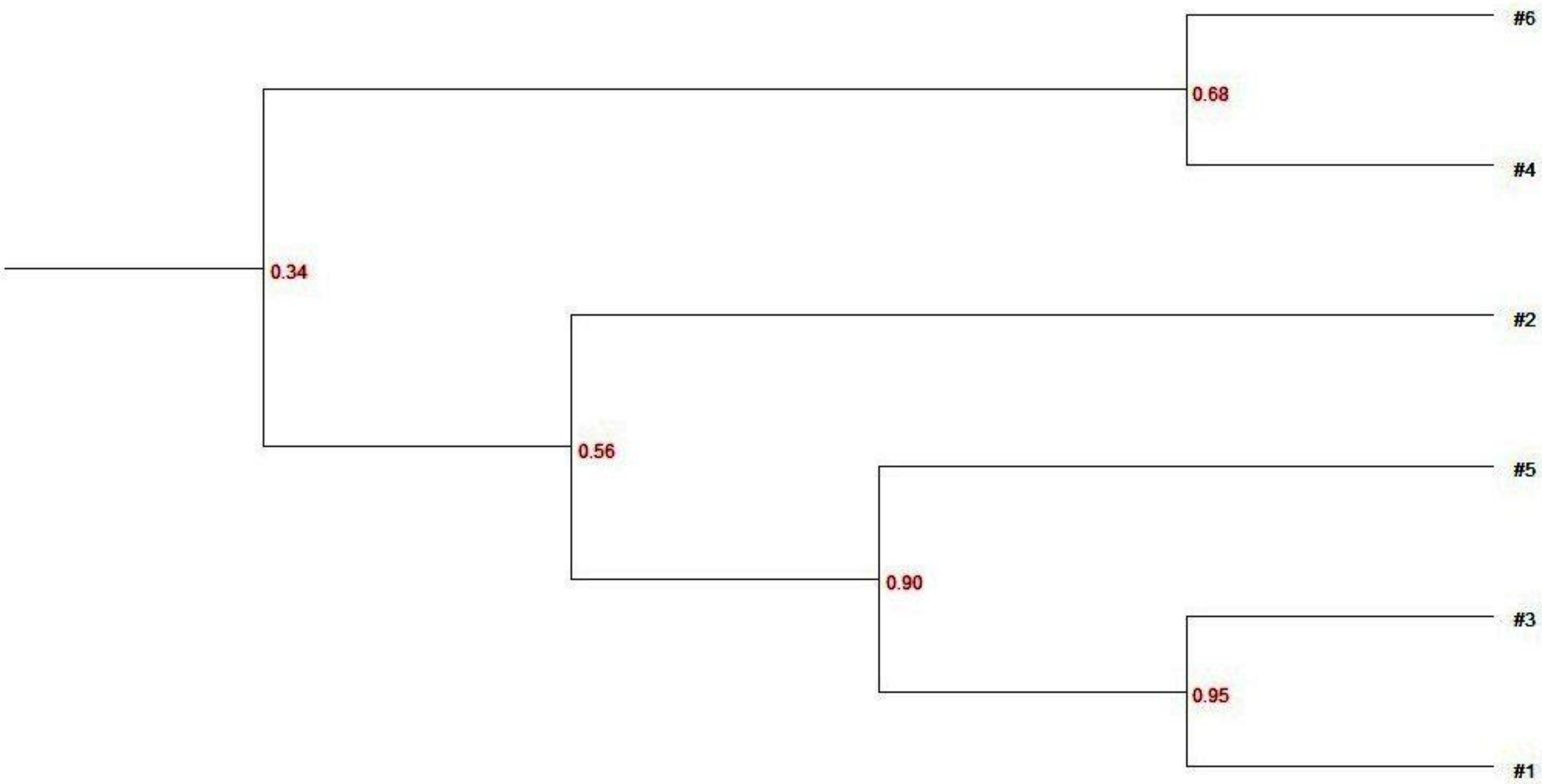

Figure S4. (b) Phylogenetic tree of electrophoretic α-esterase (α-EST) isoenzyme pattern (unprocessed image).

1.

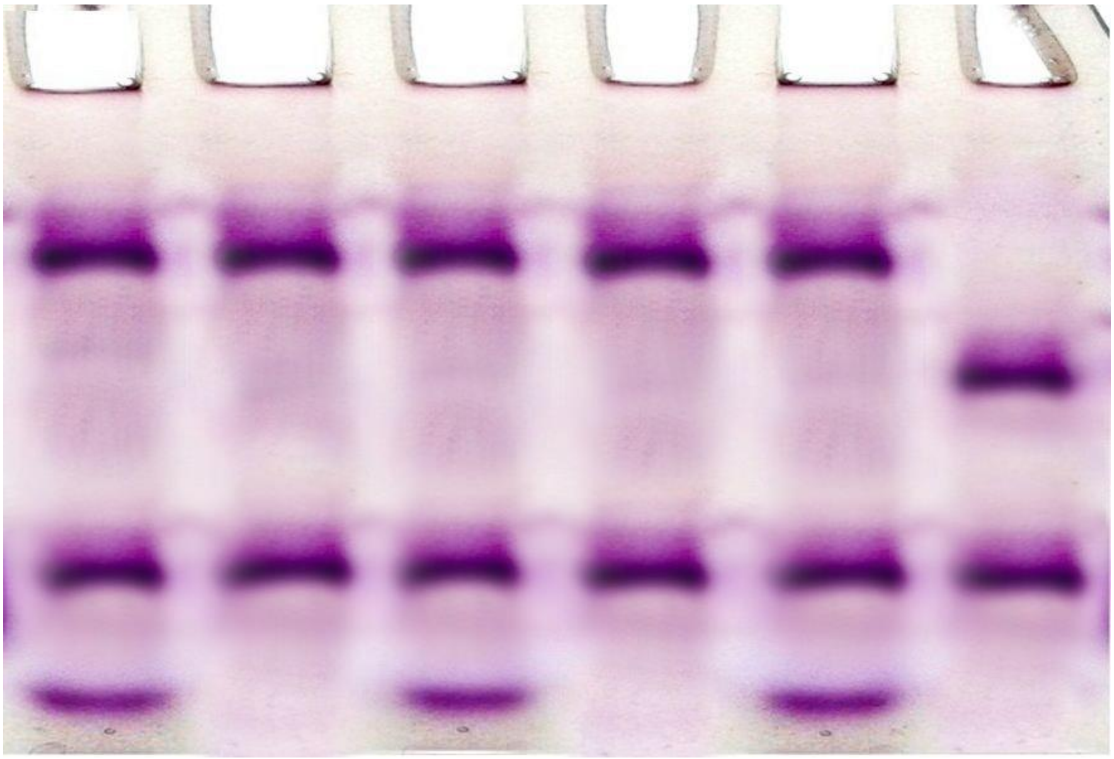

2.

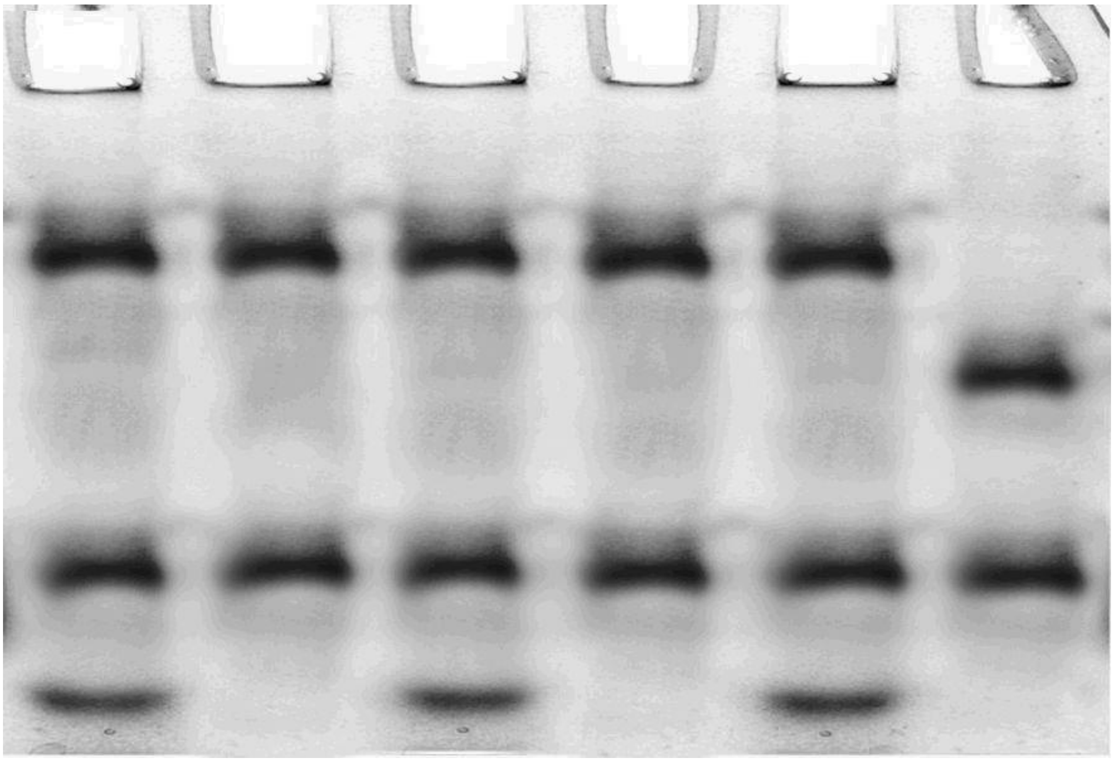

3.

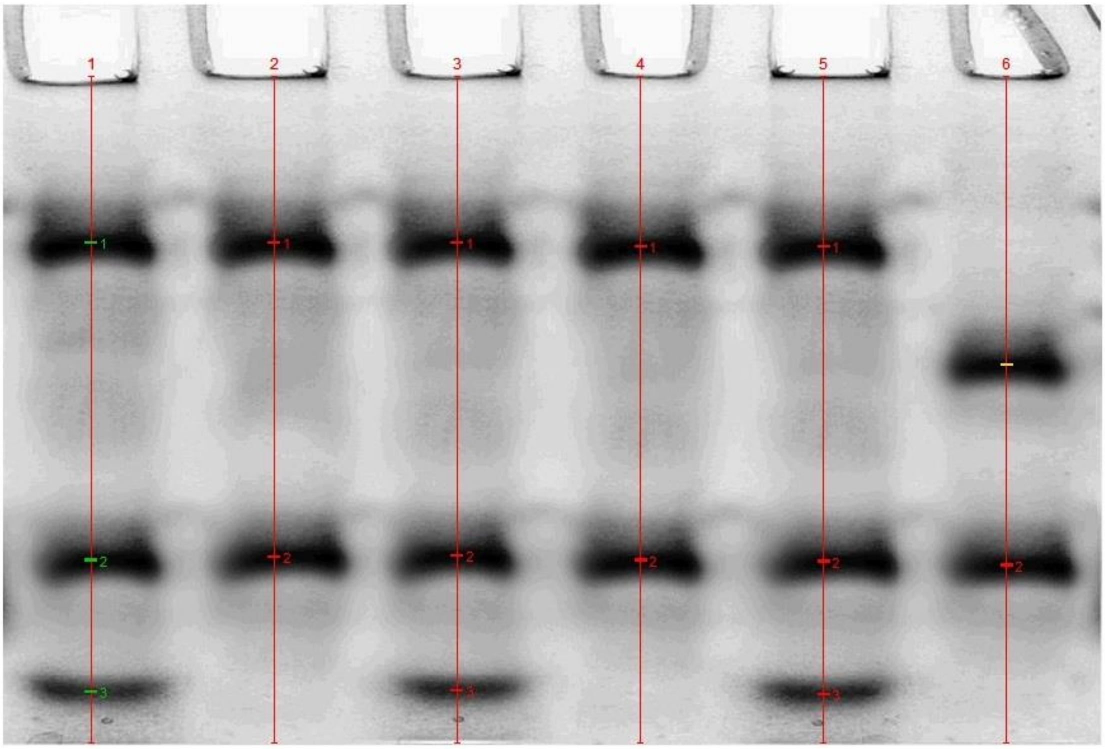

Figure S5. (a) Electrophoretic β-esterase (β-EST) isoenzyme pattern 1. unprocessed image; 2. converted image; 3. unprocessed analyzed image.

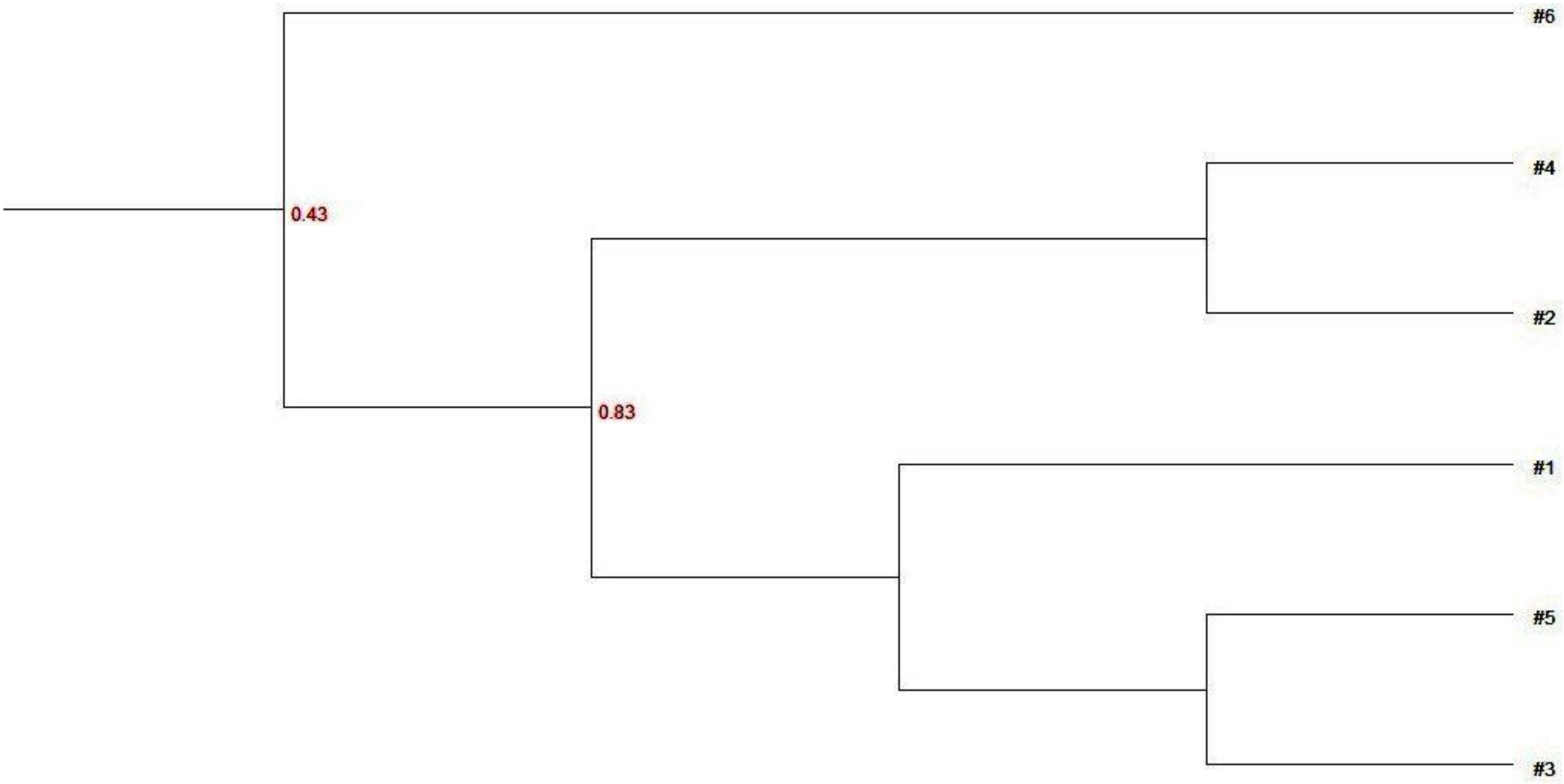

Figure S5. (b) Phylogenetic tree of electrophoretic β-esterase (β-EST) isoenzyme pattern (unprocessed image).

Table S1: Chemical analysis of protein supplements was used in the current study.

Parameter		Protein supplement
Total carbohydrate (%)		70.744
Proteins (Ug/ml)		452.33
Total lipids (Ug/ml)		8.741
Vitamin C (ng/ml)		192.47
Minerals	Potassium (K) (mm/L)	27.70
	Phosphorus (P) (mm/L)	1.42

Table S2: Mophometric characteristics of hypopharyngeal gland (Length, width (µm), surface area , and number per 10µm lobules in worker honeybees at the end of winter.

The Acini cells of the hypopharyngeal gland	Workers age	Apiary I (supplemented group)	Apiary II (control group)	Mean	F value	L.S. D
Length (µm)	New emerged	2.1^{a}±0.05	1.73^{ab}±0.14	1.92^{A}±0.11	1.0	0.278
	Nurse	1.8^{ab}±0.15	2.00^{ab}±0.05	1.90^{A}±0.08		
	Forager	1.8^{ab}±0.15	1.7^{b}±0.15	1.75^{A}±0.09		
	Mean	1.90^{A}± 0.08	1.81^{A}±0.07			
	F value	0.7			2.4	0.39
	L.S. D	0.227				
Width (µm)	New emerged	1.10^{a}±0.05	1.10^{a}±0.05	1.10^{A}±0.04	1.05	0.29
	Nurse	1.00^{a}±0.17	1.13^{a}±0.18	1.07^{A}±0.12		
	Forager	1.00^{a}±0.15	0.83^{a}±0.12	0.92^{A}±0.09		
	Mean	1.03^{A}±0.07	1.02^{A}±0.08			
	F value	0.01			0.62	0.415

	L.S. D	0.239				
Surface area of Acini cell (μm^2)	New emerged	$7.23^{a}\pm0.18$	$6.02^{ab}\pm0.76$	$6.63^{A}\pm0.44$	1.75	1.948
	Nurse	$5.62^{ab}\pm1.07$	$7.11^{a}\pm1.15$	$6.36^{A}\pm0.78$		
	Forager	$5.79^{ab}\pm1.07$	$4.34^{b}\pm0.36$	$5.06^{A}\pm0.66$		
	Mean	$6.21^{A}\pm0.54$	$5.82^{A}\pm0.57$			
	F value	0.29			1.66	2.756
	L.S. D	1.59				
Mean numbers in 10μm lobules	New emerged	$15.33^{c}\pm0.33$	$15.67^{c}\pm0.88$	$15.50^{B}\pm0.43$	10.26	3.55
	Nurse	$23.33^{a}\pm1.76$	$22.00^{ab}\pm1.52$	$22.67^{A}\pm1.09$		
	Forager	$17.00^{bc}\pm2.88$	$18.00^{bc}\pm1.15$	$17.50^{B}\pm1.41$		
	Mean	$18.55^{A}\pm1.56$	$18.56^{A}\pm1.11$			
	F value	0.00			0.27	5.03
	L.S. D	-				

Where: Apiary I:Fed on YCPC supplementary diet (Brewer's yeast, chickpea, and pollen cake), Apiary II: Control group that did not receive any supplementary diet.

Means followed by different letters within the same column or row are significantly different at $P \leq 0.05$.

Table S3: Longitudinal and transversal (μm) measurements of the second wax gland mirror of honeybee workers at the end of winter**.**

Second wax gland mirror dimensions	Workers age	Apiary I (supplemented group)	Apiary II (control group)	Mean	F value	L.S. D
Longitudinal (μm)	New emerged	47.50^{ab}±0.43	49.08^{a}±0.46	48.29^{A}±0.45	4.38	1.29
	Nurse	47.83^{ab}±0.22	49.00^{a}±0.52	48.42^{A}±0.36		
	Forager	46.00^{b}±1.14	47.67^{ab}±0.30	46.83^{B}±0.64		
	Mean	47.11^{B}±0.45	48.58^{A}±0.31			
	F value	9.18			0.10	1.83
	L.S. D	1.05				
Transversal (μm)	New emerged	15.00^{ab}±0.72	15.83^{a}±0.41	15.41^{A}±0.41	4.94	0.84
	Nurse	15.08^{ab}±0.08	15.17^{ab}±0.16	15.12^{A}±0.08		
	Forager	14.5^{b}±0.08	14.00^{b}±0.1	14.25^{B}±0.21		

		4		
Mean	14.86^{A}±0.25	15.00^{A}±0.30		
F value	0.19		1.50	1.19
L.S. D	0.68			

Where: Apiary I: **F**ed on YCPC supplementary diet (Brewer's yeast, chickpea, and pollen cake), Apiary II**:** Control group that did not receive any supplementary diet. Means followed by different letters within the same column or row are significantly different at P≤ 0.05.

Table S4: Morphometric measurements (μm) of the stinging apparatus and poison sac in honeybee worker at the end of winter.

Measuring parameters	Workers age	Apiary I (supplemented group)	Apiary II (control group)	Mean	F value	L.S. D
Sting length (μm)	New emerged	$13.17^{b}\pm0.63$	$14.25^{ab}\pm0.66$	$13.71^{B}\pm0.33$	4.59	0.91
	Nurse	$15.25^{a}\pm1.15$	$14.42^{ab}\pm0.52$	$14.83^{A}\pm0.37$		
	Forager	$14.83^{a}\pm0.76$	$14.75^{a}\pm0.43$	$14.79^{A}\pm0.22$		
	Mean	$14.42^{A}\pm0.40$	$14.47^{A}\pm0.17$			
	F value	0.03			2.63	1.29
	L.S. D	0.74				
Longitudinal dimension of poison sac (μm)	New emerged	$18.33^{c}\pm2.60$	$20.00^{bc}\pm1.25$	$19.16^{B}\pm0.83$	15.78	1.69
	Nurse	$21.67^{ab}\pm1.04$	$22.75^{a}\pm0.43$	$22.21^{A}\pm0.37$		
	Forager	$23.50^{a}\pm0.90$	$23.50^{a}\pm0.72$	$23.50^{A}\pm0.30$		

	Mean	21.17^{A}±0.90	22.08^{A}±0.57			
	F value	1.83			0.72	2.40
	L.S. D	1.38				
Transversal dimension of poison sac (μm)	New emerged	6.67^{c}±1.90	9.00^{b}±0.87	7.83^{B}±0.75	10.72	1.34
	Nurse	10.08^{ab}±0.38	9.92^{ab}±1.26	10.00^{A}±0.34		
	Forager	10.08^{ab}±0.76	11.00^{a}±0.43	10.54^{A}±0.30		
	Mean	8.94^{A}±0.66	9.97^{A}±0.39			
	F value	4.14			2.05	1.90
	L.S. D	1.10				

Where: Apiary I: Fed on YCPC supplementary diet (Brewer's yeast, chickpea, and pollen cake), Apiary II**:** Control group that did not receive any supplementary diet. Means followed by different letters within the same column or row are significantly different at P≤ 0.05.

Table S5: Biochemical parameters in homogenates of newly emerged, nurse, and forager honeybee workers in supplemented and control groups at the end of winter.

Biochemical parameters	Workers age	Apiary I supplemented group	Apiary II control group	Mean	F value	L.S.D
Total soluble proteins (mg/gb.wt)	New emerged	$11.98^{a}\pm0.59$	$1.62^{b}\pm0.07$	$6.79^{A}\pm2.33$	6.22	3.53
	Nurse	$11.86^{a}\pm3.59$	$4.20^{b}\pm0.53$	$8.03^{A}\pm2.35$		
	Forager	$4.55^{b}\pm1.48$	$0.6^{b}\pm0.20$	$2.57^{B}\pm1.11$		
	Mean	$9.46^{A}\pm1.67$	$2.14^{B}\pm0.56$			
	F value	30.57			1.97	4.99
	L.S. D	2.88				
Total carbohydrates (mg/gb.wt)	New emerged	$3.70^{b}\pm0.41$	$6.68^{b}\pm0.46$	$5.19^{B}\pm 0.72$	24.20	2.44
	Nurse	$6.83^{b}\pm 0.30$	$7.12^{b}\pm0.40$	$6.97^{B}\pm0.23$		
	Forager	$12.85^{a}\pm2.22$	$12.45^{a}\pm1.38$	$12.65^{A}\pm1.17$		
	Mean	$7.79^{A}\pm1.49$	$8.75^{A}\pm1.02$			
	F value	1.09			1.27	3.45
	L.S. D	1.99				
Total lipids (mg/gb.wt)	New emerged	$98.85^{d}\pm17.56$	$203.94^{abc}\pm27.35$	$151.39^{B}\pm27.63$	3.48	48.49
	Nurse	$148.00^{cd}\pm14.77$	$259.33^{a}\pm31.47$	$203.67^{A}\pm29.35$		
	Forager	$216.67^{ab}\pm14.$	$184.65^{bc}\pm22.$	$200.66^{A}\pm13.$		

	r	40	33	87		
	Mean	154.51^{B}±18.78	215.97^{A}±17.65			
	F value	11.44			6.63	68.57
	L.S. D	39.59				
Acetyl cholinesterase (U/gb.wt.)	New emerged	58.31^{a}±11.08	48.76^{a}±8.99	53.53^{A}±6.73	26.89	14.50
	Nurse	25.37^{b}±6.54	24.28^{b}±4.36	24.82^{B}±3.52		
	Forager	4.94^{b}±0.12	5.04^{b}±0.50	4.99^{C}±0.23		
	Mean	29.54^{A}±8.61	26.02^{A}±6.95			
	F value	0.42			0.31	20.50
	L.S. D	11.84				

Where: Apiary I:Fed on YCPC supplementary diet (Brewer's yeast, chickpea, and pollen cake), Apiary II**:**Control group that did not receive any supplementary diet. Means followed by different letters within the same column or row are significantly different at $P \leq 0.05$.
